# Functional and Nutraceutical Compounds of Tomatoes as Affected by Agronomic Practices, Postharvest Management, and Processing Methods: A Mini Review

**DOI:** 10.3389/fnut.2022.868492

**Published:** 2022-04-06

**Authors:** Giuseppina Pace Pereira Lima, Héctor Alonzo Gómez Gómez, Santino Seabra Junior, Marcelo Maraschin, Marco Antonio Tecchio, Cristine Vanz Borges

**Affiliations:** ^1^Laboratory of Plant Biochemistry, Institute of Biosciences, São Paulo State University (UNESP), Botucatu, Brazil; ^2^Academic Department of Food, Faculty of Technological Sciences, National University of Agriculture, Catacamas, Honduras; ^3^Department of Agronomy, Mato Grosso State University (UNEMAT), Nova Mutum, Brazil; ^4^Plant Morphogenesis and Biochemistry Laboratory, Federal University of Santa Catarina (UFSC), Florianópolis, Brazil; ^5^Department of Horticulture, School of Agriculture, São Paulo State University (UNESP), Botucatu, Brazil; ^6^Department of Health Sciences, Universidade Alto Vale do Rio do Peixe (UNIARP), Caçador, Brazil

**Keywords:** carotenoids, phenolic compounds, biogenic amines, postharvest, waste, by-products

## Abstract

Tomatoes and their by-products are indisputable sources of substances with antioxidants properties. Several factors limit the production and influence the nutritional and antioxidant quality of tomato fruit. However, consumers can benefit from the effects of environmental factors, such as water and hydric stress, UV radiation, agronomic practices, among others, which lead to changes in the content of secondary metabolites in tomatoes. Molecules as phenolic compounds, carotenoids, and biogenic amines are often formed in response to environmental adversities. In this way, the consumption of tomato fruits or their by-products with higher levels of antioxidants may be important adjuvants in the prevention or reduction of diseases. In this mini-review, we will present how pre- and postharvest conditions may influence the content of some bioactive compounds in tomatoes. Furthermore, we will present how some heat processing methods may change the antioxidant content, as well as, the functional and nutritional properties of the final product.

## Introduction

Tomatoes are widely used for food and beverages. The tomato crop is the largest vegetable crop in the world after potatoes and sweet potatoes. Tomatoes are an excellent source of nutrients and bioactive compounds important for human health, including phenolic compounds and carotenoids. Factors, including genetics, environmental conditions, ripeness, and postharvest conditions may influence the chemical composition and levels of phytochemicals (1).

**Figure 1:**
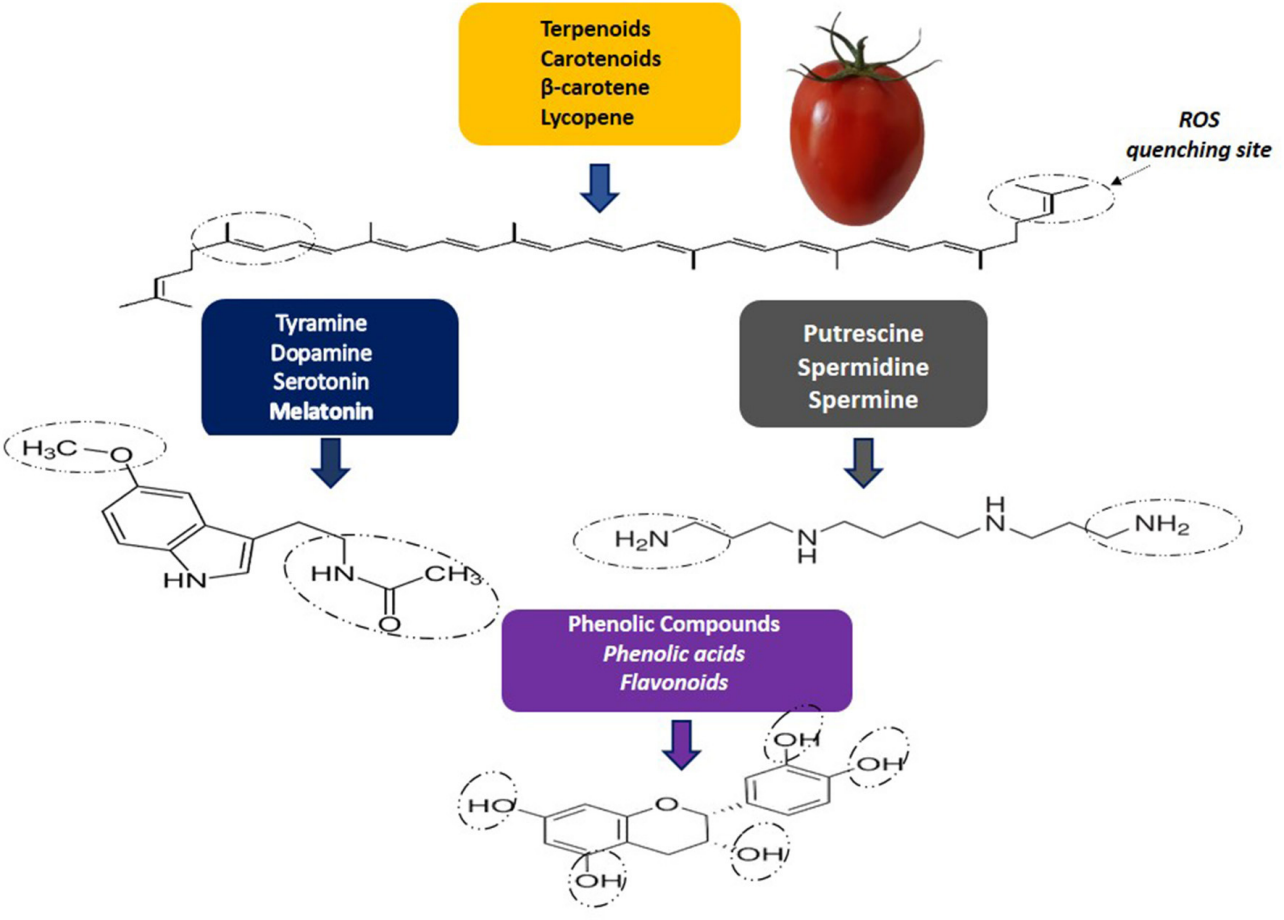
Reactive oxygen species (ROS) by phytochemical quenching.

Carotenoids, phenolic compounds and nitrogen-containing compounds are classified as non-nutritive phytochemicals. Carotenoids have a structure consisting of 40-carbon (C-40) isoprene units. Isoprenoids constitute a very representative group, which includes lycopene, β-carotene, g-carotene, z-carotene, among others (2) (Figure 1). Lycopene is responsible for the red color, and it has higher singlet oxygen quenching potential than β-carotene and α-tocopherol (3).

**Figure 2:**
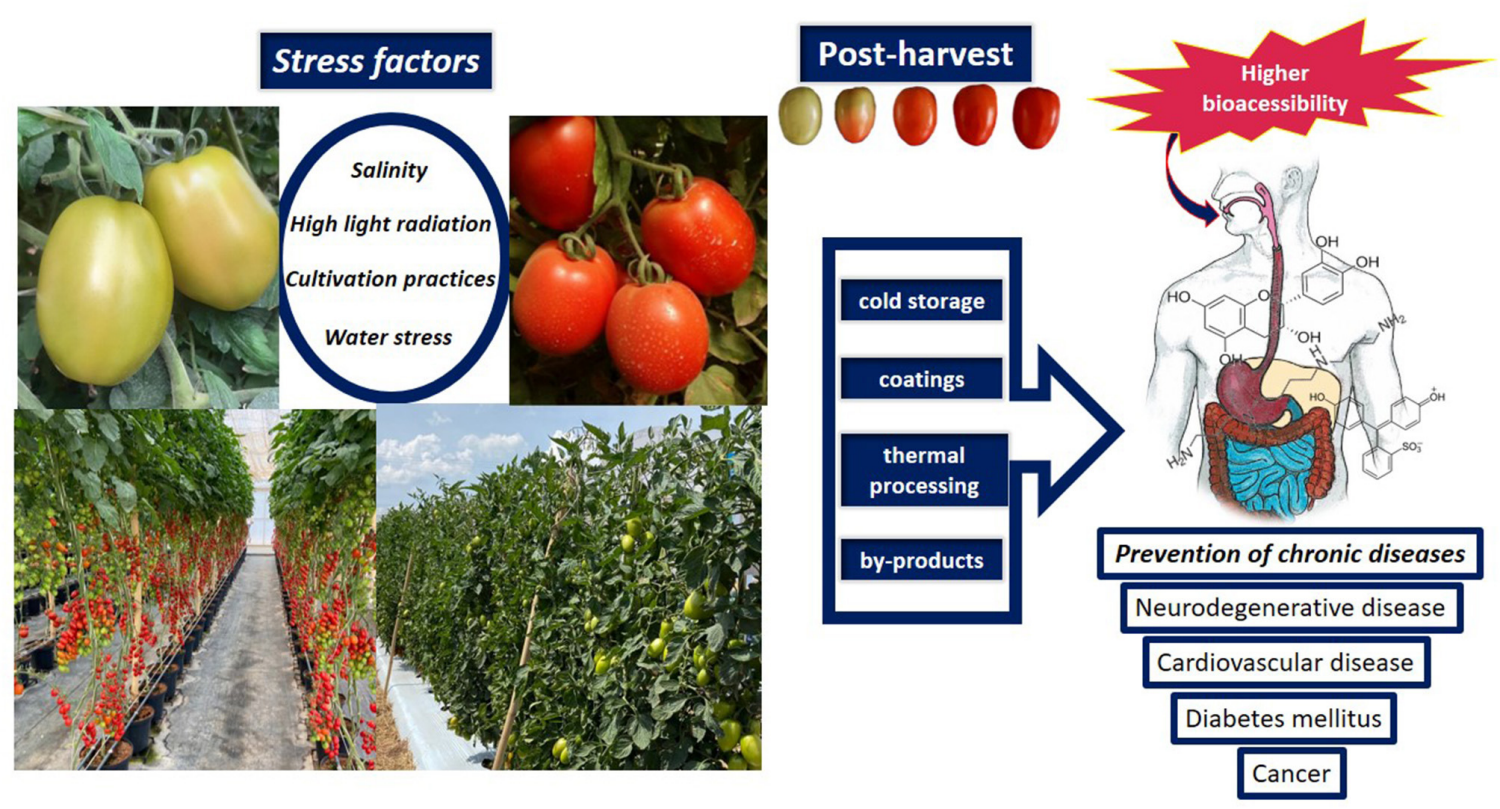
Influence of biotic and abiotic factors on the bioaccessibility and bioavailability of tomato bioactive compounds.

Phenolic compounds have one or more aromatic rings with one or more hydroxyl groups and during ripening, chlorogenic acid and quercetin have been described as major compounds in tomatoes. In ripened tomato fruit, naringenin chalcone was described as the major polyphenol (4), while caffeic acid is present in higher levels in red and yellow tomatoes (5). The content and profile of phenolic compounds are different in relation to tomato morphology, that is, phenolic acids are found in all tissues, while flavonoids and derivatives are described in the epidermis (6). The consumption of phenolic compounds is important for health, as they act as anti-atherogenic, anti-inflammatory, anti-allergenic, cardioprotective compounds (7) (Figure 2).

Biogenic amines (BAs) stand out for their antioxidant activity (Figure 1). Some Bas, as spermidine (Spd) and spermine (Spm), can play a protective role against different stresses and are related to the shelf life and quality of tomato fruits (8). Monoamines such as serotonin have also been detected in tomatoes and it was shown content ranging from 132.47 to 266.87 mg/kg, depending on the genotype (9). However, BAs in foods can also have negative impacts on health, especially due to histamine (His) and tyramine (Tyr) content. In tomatoes, His and Tyr may occur at levels ranging from not detected to 17.1 mg/kg and not detected to 6.38 mg/kg, respectively (10). Both His and Tyr were detected in cherry tomatoes but at levels below those considered harmful (His: 0.16–2.26 mg/kg; Tyr: 1.13–1.82 mg/kg) (11). Although other BAs, such as putrescine and cadaverine, do not have a direct toxic effect, they can potentiate the effects of tyramine and histamine, competing with detoxifying enzymes, in addition to being related to the formation of nitrosamines (12).

## Preharvest Factors

The environment in which the crop grows, the relative humidity, temperature, photoperiod, irradiance, soil, and water in combination with seasonal changes play a definitive role in determining the content and the profile of phytochemicals (Figure 2). Preharvest factor may promote changes in the postharvest quality in tomatoes. The selection of tomato genotypes with higher nutritional and phytochemical quality can be obtained as a function of the genotype-environment interaction (13). In contrast, in “TY Megaton” and “Yureka,” there was a reduction of phenolic compounds at the vine- and postharvest-ripened red stages (14). Variations in the profile of phenolic compounds can also occur under vine- and postharvest-ripening conditions, as well as the concentration of some specific phenolic compounds are dependent on the cultivar (15). In hydroponically grown cherry tomatoes ripened from breaker stage there was a rapid decrease in the flavonoids content (55%) compared to on-vine red ripe (16). Furthermore, the level of antioxidant compounds is different depending on the stage of maturity. After the breaker stage, the lycopene content increases reaching maximum values at the ripe-red stage, depending on the cultivar (17).

UV radiation can influence the content of phytochemicals with high antioxidant potential. UV-B (280–315 nm) induces DNA damage and leads to the production of reactive oxygen species. As a defense, the plant may also trigger the production of antioxidant compounds (18). UV-B can alter the citric acid cycle, an important pathway that provides the substrate for the synthesis of phenolic compounds through the phenylpropanoid pathway (19). In greenhouses, UV-B is usually filtered through plastics (opaque to incident UV-B). UV exclusion under a polyvinylchloride (PVC) cover may be responsible for low levels of phenolic compounds in fruits produced in greenhouses (20). Tomatoes grown under a covering that allowed solar UV transmission accumulated more phenols than those grown under a covering material that excluded solar UV (21). In tomatoes grown with doses of supplemental radiation, there was a stimulation of rutin content. However, the use of UV-A or UV-A+B did not influence other phenolic compounds or carotenoids (22).

The temperature during cultivation is a key factor for the levels of some bio-actives. Temperatures during cultivation above 30°C and below 12°C may influence lycopene levels. At higher temperatures, inhibition of lycopene formation can occur and increase the synthesis of other carotenoids, changing the color of the fruits which become more yellow (21, 22). During tomato fruit ripening in high temperature conditions (>30°C) there is an increase in rutin and caffeic acid glucoside (23). In thermotolerant saladette-type tomatoes cultivated at high temperature and in protected cultivation (24), detected high levels of lycopene (up to 2.73 mg/100 g) and β-carotene (1.33 mg/100 g). The consumption of some genotypes may amount to 54.6 and 66.5% of the daily needs of lycopene and β-carotene, considering the minimum recommended (2 mg/day) (25, 26).

Variations in vapor pressure (VP) can affect the growth, flowering and quality of tomato fruits. Low VP (deficit) results in a decrease in growth, a consequence of increased transpiration, in addition to variations in the water potential of the stem, affecting the xylem influx. VP deficit may induce fruit cracking due to the variations in fruit growth and water influx and high VP reduce fruit cracking and promote blossom-end-rot (26). Water stress also directly influences the content of secondary metabolites. It is well-argued that climate change will contribute to increasing water scarcity, and available water resources will need to be used more efficiently. In water deficit, plants tend to present an increase in reactive oxygen species (ROS) and inactivation of enzymes. In water-stressed tomatoes, it was described a decrease in lycopene and β-carotene levels and an increase in chlorogenic acid (27).

Salt stress is currently one of the biggest problems. This occurs due to a lack of water, and it may be a cause of water deficit. In salinity tolerant tomato landraces, there may be an increase in the content of phenolic compounds and carotenoids, as there was an adaptation to the saline condition (28). In saline stress, the increase in the levels of some compounds is due to the osmoregulatory/osmo-protective effect, in addition to scavenging ROS or reactive nitrogen species (RNS) formed in response to the overproduction of free radicals. BAs can scavenge free radicals, acting as membrane protectors against lipid peroxidation and oxidative stress (29). Tomatoes supplied with 80 or 160 mM NaCl and harvested at the ripe stage showed an increase in serotonin (radical scavenging activity) with average of 6.4 μg/g f.w. (30). In response to water stress, Spd and Spm levels increase in un-grafted, grafted, and self-grafted tomato fruits (31). Depending on the genotype, the content ranged from 0.55 to 2.18 mg/g d.w. Spd and 0.18 to 0.95 mg/g d.w. Spm. In water stress-resistant cherry tomatoes, increase in Spd and Spm levels were related to drought tolerance (32). The increase in Spm may be related to the osmotic potential, stomatal opening and closing, or the stimulation of NO production as a way to mitigate stress (33). Spd is described by having protective effect against cancer and metabolic and neurodegenerative disorders (34) and it has been described as delaying aging in humans (35). It is a “caloric restriction mimetic,” inducing biochemical changes similar to caloric restrictions (34). Also, it competes with acetyl CoA for binding to the catalytic site of EP300, decreasing its activity (36) and demonstrating the anticoagulant action of Spd (37).

The content of Spd, Spm, His, and total BAs content were highest in organically grown tomatoes. Hydroponically cultivated cherry tomatoes showed higher levels of phenolic compounds (817.66 mg/kg), total BAs (415.31 mg/Kg), and BAs index (BAI) (applied to determine the loss in quality) (6.63) when compared to fruits from conventional cultivation (total BA = 409.28 mg/Kg and BAI = 1.20) and to organic cultivation (total BA = 186.62 mg/kg and BAI = 0.73) (11). Regardless of the type of cultivation, serotonin was the major BAs, ranging between 167.41 to 385.96 mg/Kg. In tomato ketchup, histamine levels are slightly elevated, i.e., 22 mg/kg (38). Comparatively, the levels of His (0.16 to 2.26 mg/kg) were below the levels considered harmful to humans (500 ppm) (39) or 25 to 50 mg of His per healthy person per meal, established using non-observed adverse effect level (NOAEL) (12). It is worth noting that food intake increases the bioavailability of His, as endogenous His is synthesized from L-histidine by enzyme-dependent histidine decarboxylation in mast cells and basophils, among others (40). When an immune or non-immune stimulus occurs, promoted by viruses or other pathogens, His is released in the body. Some studies suggest that the use of antihistamines may be useful in the treatment of patients with severe acute respiratory syndrome coronavirus 2 (SARS-CoV-2) (41). The levels of Tyr in tomatoes do not appear to be harmful to human health. Studies describe levels between 0.12 to 0.18 gm/100 g (42), while other describe contents ranging from not detected to 0.31 mg/100 g (43). For a safe diet, Tyr levels should not exceed 600 mg for healthy individuals. For those who are taking a monoamine oxidase inhibitor (MAOI), levels should not exceed 50 mg (MAOI—third generation) and 6 mg for people taking classical MAOI (10).

## Postharvest Factors

### Storage and Postharvest Treatments

Removal of field heat is important as it allows longer shelf life of tomato fruits (Figure 2), as well as storage at low temperatures (4 to 12^o^C) enhance shelf-life and maintains the nutritional quality (44). Frost/freezing and chilling injuries should be avoided as they promote damage, such as low lycopene (45) and high putrescine content (46), the formation of ice crystals and damage to cell integrity, blotchy coloration (dependent on temperature and exposure time), in addition to phenolic oxidation induced by the release of polyphenoloxidase of vacuole. Storage at low or room temperatures did not influence the serotonin content (30), while exogenous melatonin in tomatoes confer chilling tolerance (47) through proline activation and NO biosynthesis (47).

The edible chitosan coating on tomato fruits induces changes in the anthocyanins and flavonoids, in addition to modifying the antioxidant capacity (48). Tomatoes treated with different concentrations of gum Arabic used as an edible coating did not show an increase in storage life, but treatment with 10% gum Arabic was efficient in maintaining the levels of phenolic compounds, carotenoids, and antioxidant activity until the 10th day of storage at 20°C (49). Due to the content and diversity of bioactives in tomatoes, they have been used as adjuvants in edible coatings. Gelatin coatings incorporated with tomato oily extracts were efficient for preserving the phytochemicals in garambullo fruits (*Myrtillocactus geometrizans*). In addition, they delayed weight loss as well as changes in pH and soluble solids, among others (50).

Natural preservative agents can be a safe option against the harmful effects of chemical residues applied in postharvest. Exogenous melatonin in postharvest may inhibit the senescence and be related to anthocyanin accumulation in ripened tomato (51), besides from inducing the endogenous melatonin levels (49, 52) and phenolic compounds (52, 53).

### Thermal Processing

Heat processing increases the bioavailability and bio-accessibility of carotenoids because, as already mentioned, there is a disintegration of the cell wall and organelle membranes where carotenoids are located (54). Heat denatures the protein-carotenoid complexes that limit the bio-accessibility of carotenoids, favoring their release from the food matrix (55). Thus, thermal processing has a direct effect on the profile and amount of carotenoids in tomatoes. The content of lycopene in raw tomatoes is ~2 mg of *t*-lycopene/g, and heating (88°C) can induce increases above 150% (52). An increase in *cis*-lycopene levels has also been described by the authors, although in a lower percentage and varying as a function of cooking time at temperatures below 100°C. At temperatures between 130 and 140°C, a decrease in *t*-lycopene content occur due to its degradation (53). It is noteworthy that in tomatoes, the bioavailability of lycopene in processed products is higher than in fresh (56).

## Tomato By-Products

Agriculture is one of the biggest generators of waste, representing millions of tons of lost and wasted food resulting from the production and sales for consumption *in natura* (57). Every year, tomato production generates a considerable amount of vegetable by-products of no commercial value, resulting from several stages which include tomato field waste before harvest and postharvest and from home or industrial processes. It is currently unthinkable that such losses could occur, given the current and future needs of the human population and the finite resources our planet has. In this sense, the use of by-products is fundamental. This is especially true for tomatoes due to their nutritional and phytochemical importance. The residues could be used for the elaboration of new products, such as flours, or be used for the production of new polysaccharides with antioxidant and anticytotoxic activity and for production of biofilms (58).

For the flour, the ideal temperature *vs*. time must be used for the drying process so that the nutritional and phytochemical quality of the product is preserved. Using field waste and tomato fruit in the late stage of production, it was found that the best drying time and temperature was 55°C for 120 min using forced convection oven (59). The study also analyzed the lyophilized product. Authors detected 11.26 μg/mg of lycopene and 162.82 l μg/mg of phenolic compounds. In the flour, kaempferol and lycopene were the major compounds detected (1.09 and 11.26 μg/mg, respectively). Among the phenolic compounds, those that appeared at the highest levels were naringerin (90.04 μg/mg) from oven-dried and catechin (255.03 μg/mg) from lyophilized by-product. In dried tomato waste, (60) detected ellagic and chlorogenic acids (143.4 and 76.3 mg/kg), and lycopene (510.6 mg/kg) and β-carotene (95.6 mg/kg) were the most abundant phenolic compounds and carotenoids. Syringic acid has been described in tomato processing by-products. It has antimicrobial activity against *Staphylococcus aureus* (61).

Phytochemicals may be unstable in response to processing conditions such as high temperatures, acidity, oxidation, light, solubility, among others that are used and it is important to study ways to protect the extraction or incorporation of bio-actives in foods to avoid loss of functionality (62). Microencapsulation has shown positive effects for incorporation of bioactive by-products in food. The addition of lycopene in spices increases the antioxidant activity and improves the stability of the bioactive (63).

## Conclusion

The beneficial effects of tomato and tomato-based products are closely related to the presence and abundance of various biologically active compounds. Agricultural practices may favor the accumulation of phytochemicals. Cultivation techniques, such as the selection of genotypes with target metabolites (e.g. high lycopene or serotonin content), temperature and vapor pressure control and water stress could be used for the production of tomatoes with superior quality and, consequently, for the development of derived products with superior amounts of beneficial compounds. Simple techniques (e.g., cooking) may facilitate the extraction and increase the bio-accessibility and bioavailability of compounds related to the reduction and/or prevention of metabolic and cardiovascular diseases and improve the functioning of the immune system. The study of management techniques in the pre- and postharvest of tomatoes, as well as the by-products generated during the productive system of the culture, becomes essential to guarantee for the population safe food with ideal quality for consumption, in addition to helping to avoid and/or reduce the frequency of food and health crises.

## Author's Note

The mechanisms involved in tomato fruit quality have been extensively investigated by physiologists and geneticists, and the responses to climatic and cultural practices have been widely described. Yet, our ability to manage and improve fruit quality in a context of global change will rely on our capacity to integrate knowledge's and anticipate interactions among genotype, environment and cultural practices.

## Author Contributions

GL and CB contributed to the conception, writing and editing of the manuscript. HG, SS, MM, and MT contributed to the writing. All authors contributed to the article and approved the submitted version.

## Conflict of Interest

The authors declare that the research was conducted in the absence of any commercial or financial relationships that could be construed as a potential conflict of interest.

## Publisher's Note

All claims expressed in this article are solely those of the authors and do not necessarily represent those of their affiliated organizations, or those of the publisher, the editors and the reviewers. Any product that may be evaluated in this article, or claim that may be made by its manufacturer, is not guaranteed or endorsed by the publisher.
